# Malignant Biliary Obstruction: Evidence for Best Practice

**DOI:** 10.1155/2016/3296801

**Published:** 2016-02-11

**Authors:** Leonardo Zorrón Cheng Tao Pu, Rajvinder Singh, Cheong Kuan Loong, Eduardo Guimarães Hourneaux de Moura

**Affiliations:** ^1^Setor de Endoscopia Gastrointestinal, Departamento de Gastroenterologia, Hospital das Clínicas da Faculdade de Medicina da Universidade de São Paulo, 05403-000 São Paulo, SP, Brazil; ^2^Gastroenterology Department, Lyell McEwen Hospital, Adelaide, Haydown Road, Elizabeth Vale, SA 5112, Australia

## Abstract

What should be done next? Is the stricture benign? Is it resectable? Should I place a stent? Which one? These are some of the questions one ponders when dealing with biliary strictures. In resectable cases, ongoing questions remain as to whether the biliary tree should be drained prior to surgery. In palliative cases, the relief of obstruction remains the main goal. Options for palliative therapy include surgical bypass, percutaneous drainage, and stenting or endoscopic stenting (transpapillary or via an endoscopic ultrasound approach). This review gathers scientific foundations behind these interventions. For operable cases, preoperative biliary drainage should not be performed unless there is evidence of cholangitis, there is delay in surgical intervention, or intense jaundice is present. For inoperable cases, transpapillary stenting after sphincterotomy is preferable over percutaneous drainage. The use of plastic stents (PS) has no benefit over Self-Expandable Metallic Stents (SEMS). In case transpapillary drainage is not possible, Endoscopic Ultrasonography- (EUS-) guided drainage is still an option over percutaneous means. There is no significant difference between the types of SEMS and its indication should be individualized.

## 1. Introduction

Neoplasms that affect the bile duct are uncommon [1–5]. Despite their rarity, estimates from the Surveillance, Epidemiology and End Results (SEER) database from North America reveal an increased incidence and a poor prognosis. The calculated prevalence in 2012 was 15 per 100,000 people [6]. It is estimated that almost 20% of the subclinical jaundice is due to malignant bile duct obstruction [7], divided by a ratio of 2 : 1 of pancreatic and other biliary obstructive cancers, respectively [8]. The most common causes of malignant biliary obstruction (MBO) are pancreatic adenocarcinoma, cholangiocarcinoma, ampullary/duodenal adenocarcinoma, gallbladder adenocarcinoma, lymphoma, and compressive metastatic proximal lymph nodes [9, 10].

Despite technological advances, only 20% of periampullary tumors are found to be resectable at the time of presentation due to their invasiveness, late symptom appearance, and onset in elderly people [11–13]. According to the Brazilian National Institute of Cancer (INCA), pancreatic tumors were accountable for 2% of the malignant tumors with an estimate of around 17,000 new cases in 2015. Having in mind that only 15%–20% of these neoplasms are resectable, the number of inoperable MBO in Brazil in the year of 2015 is estimated to be about 13,000 patients [14].

Biliary tree obstruction and consequent jaundice occur in 70–90% of these patients and have important consequences mainly for the patient's quality of life, morbidity, and overall mortality [15–19]. Options for palliative therapy of biliary tree obstruction include surgical bypass, percutaneous external drainage/stenting, and endoscopic stenting. For patients with resectable tumors, ongoing debate remains on whether preoperative drainage is necessary. Commonly, MBO appears as painless jaundice with anorexia and weight loss. This can sometimes be present in other benign conditions (i.e., chronic pancreatitis) [9, 10]. Although the diagnosis can be achieved without tissue biopsy, it is important to have histological confirmation. Tissue can be acquired through interventional radiology (ultrasound/computed tomography-guided puncture) or through endoscopic procedures, such as Endoscopic Retrograde Cholangiopancreatography (ERCP) and Endoscopic Ultrasound-Guided Fine-Needle Aspiration (EUS-FNA), although the former can be associated with seeding in the needle tract [20–23].

To further evaluate a biliary stricture, it may be necessary to perform a computed tomography (CT) scan or a Magnetic Resonance Cholangiopancreatography (MRCP). If a mass is observed, a tissue sample should be obtained via the methods previously mentioned. If no mass is seen, an EUS should be performed and any visualized lesion can be sampled. ERCP with brush cytology, cholangioscopy, endomicroscopy, and/or intraductal ultrasound can be performed to further evaluate this [9, 10, 24].

The European Society of Gastrointestinal Endoscopy's (ESGE) guidelines [25] recommend placing a plastic stent for biliary drainage if a diagnosis of the biliary obstruction (malignant versus benign) is still not ascertained. Despite this recommendation, patients with a high clinical-imaginologic suspicion of MBO (i.e., an elderly male patient, smoker, with high bilirubin levels, anorexia, and metastatic disease on imaging) could benefit in using a fully covered Self-Expandable Metallic Stent (cSEMS), therefore avoiding the cost and possible complications of another ERCP. The current practice however is to place a plastic stent (PS) in cases of cholangitis as there is little data about the possible advantages of SEMS in these cases.

This review intends to explore the possibilities of drainage of the biliary tree in patients with malignant biliary obstruction.

## 2. Operable Cases

### 2.1. Preoperative Drainage

The routine use of preoperative biliary drainage (PBD) is not well defined yet, although its use in cholangitis, neoadjuvant therapies, and delayed surgery is advocated by the ESGE.

A meta-analysis from Cochrane [26], involving six randomized controlled trials (RCTs) with percutaneous and endoscopic interventions, revealed major morbidity and no change in mortality in the group who was subjected to preoperative drainage. However the clinical status of patients were rather heterogeneous amongst the studies and the stent used was PS. There is scarce material regarding the use of Self-Expandable Metallic Stents (SEMS) for PBD.

A retrospective study published in January 2015 [27] evaluated the use of percutaneous transhepatic biliary drainage (PTBD) versus SEMS versus PS versus no drainage in PBD. The results demonstrated a significantly higher rate of sterile bile in the no drainage group, although this finding was not translated in less infection in the postprocedure period. There were no differences between SEMS and PS. Nonetheless, the sample had a small percentage of SEMS placed (15%) and the median prestent bilirubin level was 201 *μ*mol/L. The severity of jaundice is used in clinical practice to infer the severity of the obstruction and is beginning to figure as an important factor to look at in recent articles. Sauvanet et al. [17] published in August 2015 an article demonstrating that a cutoff value of 250–300 *μ*mol/L in serum bilirubin level had clinical impact in patients with recurrent jaundice after pancreaticoduodenectomy due to pancreatic adenocarcinoma, and patients above this cutoff value had higher morbidity and mortality.

A RCT published in August 2015 added information not only regarding drainage or not but also analyzing the types of drainage. It revealed an overall complication rate for cSEMS, PS, and early surgery groups of 51%, 74%, and 39%, respectively [28]. The evidence gathered so far in the use of preoperative drainage indicates that it can be specifically beneficial also in proximal obstructions and there may be a better outcome if SEMS are used [29, 30] in this tendency. More studies are needed to determine a possible benefit in patients with high bilirubin levels and long wait for surgery (either for logistic reasons or for need of clinical compensation/neoadjuvant chemoradiation), as well as the neoplasia's topography most benefited by the drainage and the optimal material for the stent. In any case, if preoperative drainage has been deemed necessary, it is needed to ponder the type of intervention: percutaneous (PTBD) or endoscopic. PTBD has higher morbidity due to the risk of puncture-related hemorrhage, cutaneous infection, and catheter tract recurrence. Percutaneous tract seeding is a major preoccupation and can compromise up to 5.2% of potentially curable cases [31]. Nonetheless, some studies have shown that, in patients with proximal tumors, the endoscopic drainage can have lower technical success (38% of cases) [32] and should preferably have an internal drainage [33]. If PTBD is chosen, sphincterotomy should be performed in transpapillary stent placement; in order to prevent pancreatitis for tumors more than 2 cm in distance from the papillae, a suprapapillary stent could be used as an option for transpapillary stenting [34, 35].

These articles show evidences supporting either therapy. Since they involved data as far as 14 years ago, their data may be not applicable for the current practices and technical success; therefore more studies regarding the type of preoperative drainage are needed for a definitive answer.

In summary, the evidence thus far reveals that, for patients with a low bilirubin level and scheduled early surgery, preoperative drainage should be avoided. On other routine PBD cases, the conflicting data suggests that individual case scenarios should be analyzed.

## 3. Inoperable Cases

### 3.1. Palliative Surgical Bypass versus Endoscopic Drainage

Although initial results with surgical bypass demonstrated low rates of recurrent jaundice (2–5%), the surgery itself carries an appreciable risk of postoperative morbidity and mortality, in up to one-fourth of the patients in some trials [36, 37]. Despite the evidence of more complications with surgical decompression, it has been advocated in patients who at the time of laparotomy for planned tumor resection are found to have unresectable disease as well as in occasional patients with longer projected survival due to its longer jaundice relief [38, 39].

In order to analyze the possible treatments for inoperable MBO, RCTs have compared some of these interventions and found that, despite a shorter time for recurrence of jaundice, the complication rate was lower in the endoscopic group [40, 41]. A recent meta-analysis from 2015 regarding surgical bypass versus endoscopic stenting for distal inoperable MBO demonstrated no differences for success of the procedures, but differences were observed with better outcomes for endoscopic therapy with 10% less mortality and 19% less complications associated with the procedure [42].

In summary, from a palliative perspective, the use of an endoscopic approach appears to be favorable.

### 3.2. SEMS versus PS

Two main types of materials for stents are routinely used in current practice: plastic and metal. Several RCTs demonstrated that SEMS are associated with longer stent patency but survival rate is quite similar to PS. Some studies favored survival in the SEMS group [43–52] while some favored the PS group [53, 54]. Statistically significant survival difference has only been shown in one study, favoring SEMS [55].

The latest meta-analysis regarding metal and plastic stenting in inoperable MBO [56], which involved stents inserted through ERCP, involved thirteen RCTs and demonstrated a better survival of about 1-2 months in the SEMS group. In this meta-analysis, the use of SEMS had 24% fewer dysfunction, almost double patency (124 days versus 250 days), and longer survival. It also required 30% fewer reinterventions, when compared to PS. Despite no statistical differences in costs and complication amongst the two stents groups, there was numerical difference benefitting SEMS (€4,193.98 for SEMS versus €4,728.65 for PS, *P* = 0.09 and 3% less complication, *P* = 0.16).

Kim et al. [57] demonstrated a survival benefit in metastatic biliary tract cancer of about 9 months in a phase II study of gemcitabine and S-1 combination chemotherapy, in contrast to the 3-4 months survival of earlier studies. This is also true for pancreatic adenocarcinoma that has a mean overall survival of 6.9 months with new treatments [58]. Therefore, taking into account the patients' quality of life and adequate palliative care with the lowest hospital stay possible and minimal symptomatology, SEMS is always the first option.

An important aspect is to look at the whole treatment cost rather than the cost of the specific instrument. Therefore, a prospective randomized controlled study with attention to a specific population (short expected survival) is needed to clarify if SEMS is actually more cost-effective in this group.

The summary of evidences presented so far points out that the use of SEMS is advisable. Even in the short expected survival cases, the question we should ask ourselves is why not use SEMS, as it does not cost more, has similar complication, and does have better outcomes [56].

### 3.3. Types of SEMS

Endoscopic stents appear to offer a less invasive option, but the many designs and stent types available have made selecting the ideal stent for individual patients complicated. There are several combinations of materials, with or without antireflux valves, uncovered SEMS (uSEMS), partially covered SEMS (pcSEMS) or cSEMS, and different kinds of mesh. All of them have different possible complications and conflicting information in the literature [59–63]. To date, two meta-analyses demonstrated no benefits to survival or morbidity in cSEMS compared to uSEMS [64, 65]. There is still no SEMS that has presented a far superior result compared to the others.

Usually uSEMS are associated with obstruction due to ingrowth while cSEMS have higher migration rates and association with cholecystitis if placed across the cystic duct in patients not cholecystomized [59, 64]. A retrospective study from 2013 evaluated uSEMS versus cSEMS and found that the adverse event rate is about 27% for both, tumor ingrowth with recurrent obstruction is more common in the uSEMS group (76% versus 9%, *P* < 0.001), and stent migration is more common in cSEMS group (36% versus 2%, *P* < 0.001) [62]. A more recent pcSEMS was developed, trying to gather the best of both worlds. Apparently, it has better results with less stent migration than cSEMS but more than uSEMS (pcSEMS 5.9% versus uSEMS 0%, *P* = 0.118) and less tumor ingrowth than uSEMS (pcSEMS 5.9% versus uSEMS 19.2%, *P* = 0.041) [63].

The major causes of dysfunction of the large bore cSEMS are attributed to the reflux of duodenal content into the prosthesis and to the stent migration. Although studies with innovative mechanisms to surpass the migration problem failed to show any difference [63], the antireflux mechanism has shown to lead to longer patency. In the study by Lee et al. [66], the overall reflux of barium was 7.7% in the Anti-Reflux Valve Metal Stent (ARVMS) group versus 100% in the cSEMS group and the cumulative median duration of stent patency was 407 days for ARVMS versus 220 days for cSEMS.

In order to overcome the main problem of obstruction due to tumor ingrowth when using the uSEMS, the use of novel SEMS that are combined with radioactive seeds (I125) or brachytherapy is still being studied and has shown promising results regarding patency time and survival (mean survival of 8 months versus 3 months in the study by Zhu et al.) [67–69]. The use of drug-eluting stents, namely, with paclitaxel, had not shown expressive benefits [70].

The use of bilateral versus unilateral SEMS in the proximal MBO is an issue not yet resolved. Despite the better cumulative patency demonstrated in the studies, the complication and survival rates do not seem to improve in bilateral drainage, although the physiologic mechanism that would lead to a better outcome seems plausible [71–73].

Altogether it is not yet possible to state the optimal choice for palliative SEMS in MBO. Hence, each case has to be assessed individually and evaluated regarding the pros and cons. Novel products and techniques are promising but lacking in RCTs in favor of certain specific SEMS.

### 3.4. Radiofrequency Ablation

There are several papers in recent years demonstrating a good outcome in patients submitted to radiofrequency ablation (RFA) after the placement of a SEMS (occluded SEMS) or before its placement [74–76]. It is usually preferred over photodynamic therapy due to its complications [77–79]. Intraductal RFA can be performed either endoscopically or percutaneously through the insertion of a specific catheter that delivers heat energy directly to neoplastic tissues to achieve tumor necrosis and to prolong biliary patency. This procedure has a local effect and is not intended to be curative.

Despite its promising results, it is an experimental therapy that just a handful of centres have at disposal. There is still much research to be done before we can reach a consensus regarding how, when, and where this new technique should be used.

### 3.5. EUS

EUS assists in accessing the biliary tract via transgastric or transduodenal routes and is an option in cases where transpapillary route is inaccessible by ERCP. It is usually performed with a sectorial echoendoscope which identifies the hepatic ducts or the bile duct. The duct is punctured and a guidewire is placed, to guide the SEMS through the gastric or duodenal wall. Alternatively, EUS can also be used to exteriorize the guidewire through the papillae to guide a usually placed SEMS through ERCP in the so-called* rendezvous* technique. Although these state-of-the-art techniques are exciting, more studies are needed to confirm their efficacy and security compared with the percutaneous option when the transpapillary ERCP drainage is not possible.

Therefore, it is mainly an option in cases of failed transpapillary endoscopic drainage, with the advantage of maintaining physiologic bile flow through the gastrointestinal tract and having better comfort to the patient as internal drainage [80, 81].

## 4. Conclusions

In operable cases, routine preoperative stents shall not be placed unless the patient is cholangitic, there is delay in surgery, or intense jaundice is present. More research is needed to clarify the benefits of PTBD in proximal tumors and the cutoff level of bilirubin. Most of the studies used PS for PTBD, with just a few studies examining the role of SEMS. Therefore more data regarding SEMS for PBD is necessary to have a definitive answer.

For inoperable cases, surgery should be avoided and transpapillary stenting after sphincterotomy should be preferable over the percutaneous drainage approach. The use of PS for MBO has no demonstrated benefit over SEMS and should not be used, since new modalities of chemotherapy for metastatic patients, either with pancreatic adenocarcinoma or with cholangiocarcinoma, surpass 6 months of mean overall survival. In inaccessible transpapillary cases, EUS-guided drainage is still an option over the percutaneous approach. Among the types of SEMS, there is no significant difference between their uses and their indication should be individualized. The concurrent use of SEMS with radioisotopes, brachytherapy, and radiofrequency ablation shows promising results.
